# Low Serum Methylglyoxal Levels Correlate with Psoriasis Severity and Inflammatory Response Indices

**DOI:** 10.3390/pathophysiology32010008

**Published:** 2025-02-03

**Authors:** Aleksandra Damasiewicz-Bodzek, Agnieszka Nowak, Maciej Maciejczyk, Sławomir Waligóra, Brygida Przywara-Chowaniec

**Affiliations:** 1 Department of Chemistry, Faculty of Medical Sciences in Zabrze, Medical University of Silesia, 40-055 Katowice, Poland; aleksandra.bodzek@sum.edu.pl (A.D.-B.); d201056@365.sum.edu.pl (M.M.); swaligora@sum.edu.pl (S.W.); 2 2nd Department of Cardiology, Faculty of Medical Sciences in Zabrze, Medical University of Silesia, 40-055 Katowice, Poland; bprzywara-chowaniec@365.sum.edu.pl

**Keywords:** psoriasis, methylglyoxal, ELISA, PASI, BSA, DLQI

## Abstract

Psoriasis is a multifactorial inflammatory disease. Methylglyoxal (MG) is a highly reactive dicarbonyl compound responsible for dicarbonyl stress in some inflammatory conditions, and it may play a role in the etiopathogenesis of psoriasis. **Methods:** A total of 50 patients with psoriasis and 35 healthy individuals participated in this study. The following indices were assessed in patients: Body Surface Area (BSA), Psoriasis Area and Severity Index (PASI), and Dermatology Life Quality Index (DLQI). MG concentration was evaluated in blood samples. The following inflammatory response indices were calculated: Systemic Inflammation Response Index (SIRI), Systemic Immuno-inflammation Index (SII), and Aggregate Index of Systemic Inflammation (AISI). **Results:** An analysis of the obtained data showed a statistically significant decrease in the mean serum MG concentration in patients with psoriasis when compared to the healthy individuals (1.19 ± 0.4 μg/mL vs. 1.75 ± 0.6 μg/mL; *p* = 0.000002). In the patients, MG concentration correlated negatively with psoriasis disease severity indicators (BSA and PASI), C-reactive protein (CRP) concentration, and inflammatory response indicators (SII and AISI). **Conclusions:** The decreased concentration of MG may be attributed to an increased accumulation of its derivatives (advanced glycation end-products) in the inflamed skin and/or scavenging by polyamines.

## 1. Introduction

Methylglyoxal (MG, also abbreviated as MGO) is a reactive α-dicarbonyl compound primarily formed in the body during the degradation of dihydroxyacetone phosphate and glyceraldehyde 3-phosphate (which are compounds involved in glycolysis, gluconeogenesis, and glycerol metabolism). Other sources of MG include advanced glycation (also known as glycoxidation or the Maillard reaction), degradation of glycated proteins, catabolism of threonine, ketone body metabolism, and oxidation of aminoacetone and acetone. Other important α-dicarbonyls that result from human metabolism include glyoxal (GO) and 3-deoxyglucosone (3DG) [1,2,3]. Dietary sources of MG are considered negligible [1], although the typical Western diet contributes to increased MG exposure [4].

MG is involved in pathological processes, such as diabetes, hypertension, and obesity [3,5]. It contributes to protein carbonylation, which can make proteins highly immunogenic [6] or cause them to lose their properties [7]. For example, altered collagen has modified mechanical properties, leading to reduced cell adhesion. Cell signaling and differentiation are disturbed in such an environment. The homeostasis of the extracellular matrix is affected, contributing to fibrosis [8]. Additionally, the proteasomal degradation of proteins incubated with MG is hindered; therefore, such damaged proteins may accumulate in tissues [9].

MG and other oxoaldehydes are detoxified by the glyoxalase system. This system consists of two enzymes—glyoxalase-1 (Glo1) and glyoxalase-2 (Glo2). Glo1 requires the cofactor glutathione (GSH). The final product of MG detoxification is D-lactate [10,11].

Certain diseases are associated with increased dicarbonyl stress. Elevated MG concentrations and/or increased amounts of MG derivatives are related to fatal cardiovascular events in patients with type 1 and type 2 diabetes, as well as neurological disorders (Alzheimer’s disease, Parkinson’s disease, cerebral atrophy, and polyneuropathies), chronic kidney disease, nonalcoholic fatty liver disease, liver steatosis, atherosclerosis, and rheumatoid arthritis [12,13].

Psoriasis is a chronic inflammatory disease with a very complex etiopathogenesis. In addition to the skin, it may affect the joints and nails, significantly decreasing quality of life. The systemic inflammation concomitant with this disease leads to other medical conditions, such as cardiovascular disease, diabetes mellitus, arterial hypertension, obesity, dyslipidemia, and others [14,15].

It is well established that oxidative stress is increased in psoriasis patients and contributes to their comorbidities [16,17,18]. As oxidative conditions are a significant factor in the glycation reaction, it can be hypothesized that psoriasis patients are also at an increased risk of dicarbonyl stress. Therefore, the aim of our study was to evaluate serum MG concentration in patients with psoriasis and analyze its correlation with disease severity and inflammation indices.

## 2. Materials and Methods

The study group consisted of 50 subjects suffering from psoriasis (30 men and 20 women, mean age 52.8 ± 14.5 years), all admitted to a dermatological ward due to an exacerbation of the disease. The mean disease duration was 17.5 ± 14.6 years (ranging from 1 month to 55 years). In two cases, it was the first clinical manifestation of the disease. Psoriatic patients with comorbidities were excluded from the study. The control group consisted of 35 healthy volunteers (15 men and 20 women) of similar age (47.5 ± 13.8 years, *p* > 0.05), with no family history of psoriasis. All participants were of Caucasian ethnicity.

Venous blood samples were collected after an overnight fast (in psoriatic patients, samples were collected before the initiation of treatment). Blood serum was obtained through centrifugation and stored at −80 °C until analysis. The study protocol was approved by the Local Bioethical Commission of Medical University of Silesia in Katowice (approval number PCN/CBN/0052/KB1/63/I/22, dated 20 September 2022), and all participants provided voluntary, informed, written consent for participation.

In patients with psoriasis, disease severity was assessed using the Body Surface Area (BSA) scale and the Psoriasis Area and Severity Index (PASI). The quality of life was assessed using the Dermatology Life Quality Index (DLQI). All indices were evaluated by the same dermatologist. Routine blood laboratory tests were also carried out in the patients. Based on the full blood count, the following inflammatory response indices were calculated: Systemic Inflammation Response Index (SIRI), Systemic Immuno-inflammation Index (SII), and Aggregate Index of Systemic Inflammation (AISI).

SIRI is defined as the ratio of [neutrophil count × monocyte count]/lymphocyte count. SII is the ratio of [neutrophil count × thrombocyte count]/lymphocyte count. AISI is the ratio of [neutrophil count × thrombocyte count × monocyte count]/lymphocyte count. These indices are novel general biomarkers of inflammation intensity, widely studied in various diseases. Recent research suggests that they may also be useful in assessing the risk and severity of psoriasis [19,20,21]. It has been proven that they are applicable in psoriasis therapy strategizing and treatment response monitoring [22,23].

Enzyme-linked immunosorbent assay (ELISA) was used to evaluate MG concentration in the samples. The commercially available OxiSelect™ Methylglyoxal (MG) Competitive ELISA Kit (Cell Biolabs, Inc., San Diego, CA, USA) was employed according to the manufacturer’s protocol.

Absorbance measurements were performed using a Multiskan FC Type 357 microplate photometer equipped with SkanIt version 7.0 software for Multiskan FC (Thermo Scientific, Waltham, MA, USA). The obtained results were presented as basic parameters of descriptive statistics: mean, standard deviation (SD), median, and minimum–maximum range.

The distribution of data was tested for normality using the Shapiro–Wilk test. Comparisons between the psoriasis and healthy control groups were performed using Student’s *t*-test and non-parametric Kolmogorov–Smirnov and Mann–Whitney U tests. To analyze correlations, Pearson’s test and Spearman’s rank correlation test were used. A *p* value of <0.05 was considered statistically significant. Statistical analyses were performed using TIBCO Statistica version 13.3 (TIBCO Software Inc., Palo Alto, CA, USA).

## 3. Results

Clinical and laboratory parameters of the examined group of patients are presented in Table 1. We found that 20% of patients had a BSA ≤ 10%, 55% had a BSA between 11 and 30%, and 25% had a BSA > 30%. Moreover, 43% of patients had a PASI ≤ 10, 43% had a PASI between 11 and 25, and 14% had a PASI > 25. Furthermore, 32% of patients had a DLQI ≤10, 50% had a DLQI between 11 and 20, and 18% had a DLQI > 20. According to the classification proposed by Salgado-Boquete et al., 52% of our patients suffered from severe psoriasis (PASI ≥ 11 and BSA ≥ 10%); 46% had moderate psoriasis (PASI ≥11 or BSA ≥ 10%, but DLQI ≥ 5); only one patient (2%) had mild psoriasis (PASI ≤ 3 or BSA ≤ 5%, and DLQI < 5) [24].

In addition, 67% of patients had CRP values within the normal range (<5 mg/L); 26% had CRP values between 5 and 15 mg/L; and 7% had CRP values higher than 15 mg/L.

**Figure 1:**
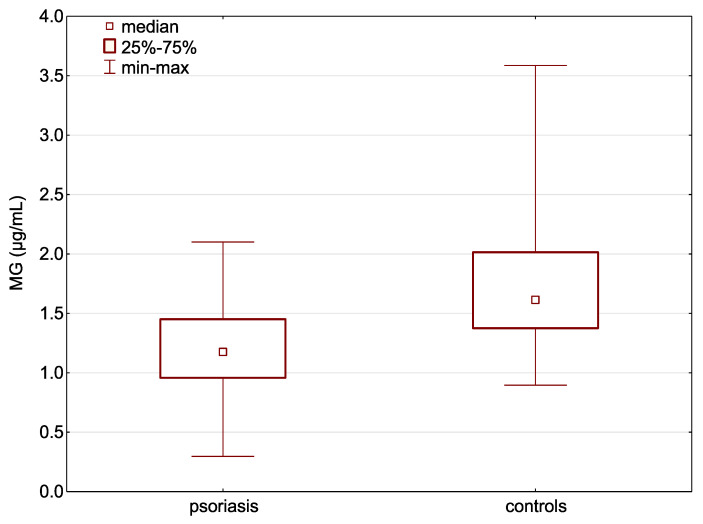
Concentrations of serum MG in patients with psoriasis and healthy individuals.

An analysis of the obtained results showed a statistically significant lower mean serum concentration of MG in patients with psoriasis compared to the healthy individuals (1.19 ± 0.4 μg/mL vs. 1.75 ± 0.6 μg/mL; *p* = 0.000002; Figure 1).

**Figure 2:**
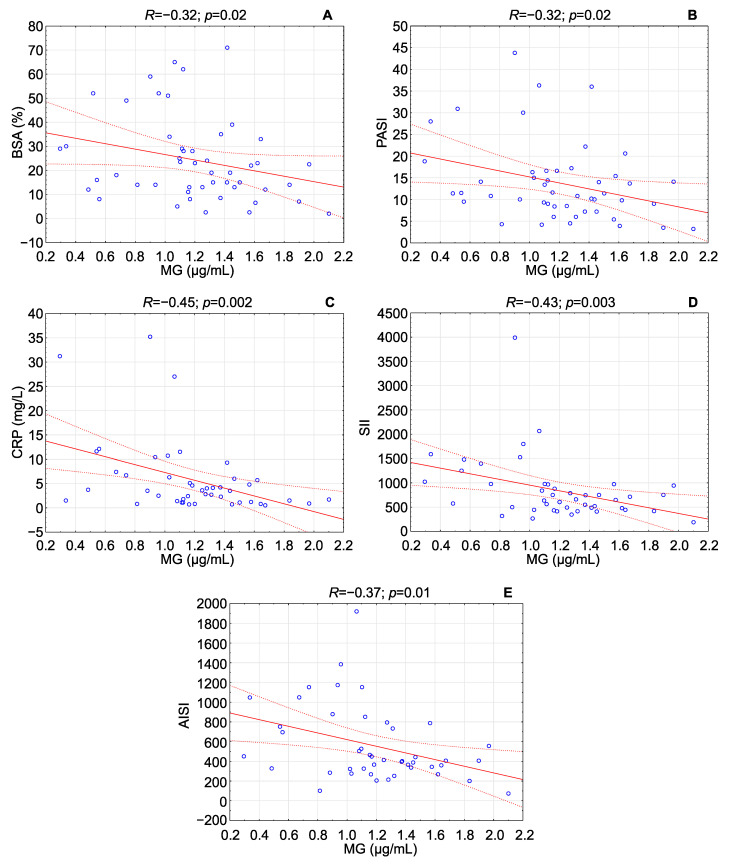
Plots depicting statistically significant correlations of MG concentration with (**A**) BSA score, (**B**) PASI score, (**C**) CRP concentration, (**D**) SII score, and (**E**) AISI score in patients with psoriasis.

In patients with psoriasis, MG concentration in serum negatively correlated with the disease severity indices BSA and PASI, CRP (C-reactive protein) concentration, and the inflammatory response indicators SII and AISI. These correlations were either weak (between −0.1 and −0.3) or moderate (between −0.4 and −0.6) [25], suggesting only partial associations between MG levels and disease indicators as well as inflammatory markers. The details are summarized in Table 2 and Figure 2.

CRP concentrations did not correlate significantly with BSA (*p* = 0.37), PASI (*p* = 0.10), DLQI (*p* = 0.28), or SIRI (*p* = 0.08). However, CRP concentrations correlated positively with leukocyte count (*p* = 0.04, *R* = 0.30), SII (*p* = 0.005, *R* = 0.43), and AISI (*p* = 0.006, *R* = 0.41). Mean MG concentration, CRP concentration, leukocyte count, SIRI, SII, and AISI did not differ significantly between patients with severe psoriasis (1.22 μg/mL; 5.04 mg/L; 8.09 × 10^9^/L; 2.26; 833.90; 604.95, respectively) and those with mild–moderate psoriasis (1.28 μg/mL; 3.63 mg/L; 7,87 × 10^9^/L; 1.91; 707.89; 491.87, respectively), with *p* values for all comparisons >0.05.

## 4. Discussion

In our study, we observed significantly lower MG concentrations in psoriasis patients compared to healthy individuals. MG concentration correlated negatively with BSA, PASI, CRP, SII, and AISI in the patients. While MG concentration was lower in the severe psoriasis group compared to the mild–moderate psoriasis group, this difference was nonsignificant.

**Figure 3:**
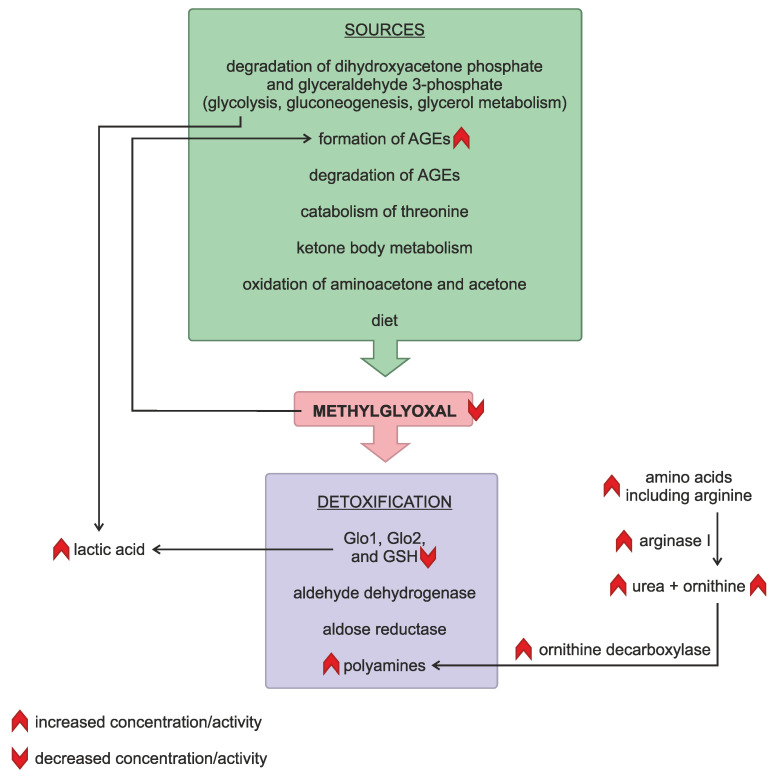
Relationships between methylglyoxal and known disturbances in metabolism in psoriatic patients.

Unfortunately, determining the overall direction of metabolic alterations in psoriasis is challenging due to the limited knowledge and complexity of these phenomena (Figure 3). It is noteworthy that 179 genes have been identified as either under- or overexpressed in psoriatic lesions [26]. Alas, we came upon only one previous study on serum MG levels in psoriasis patients [16]. To our surprise, the results were contradictory to ours: significantly higher MG levels were detected in the patients. However, the examined group consisted of only 20 subjects with psoriasis and 10 healthy individuals (from an initial pool of 60 and 47 participants, respectively). Moreover, Kaur et al. focused on the relationship between MG levels and oxidative stress parameters [16], whereas in our study, in addition to psoriasis severity and activity scores, we analyzed systemic inflammation indices. In our study, MG correlated negatively with PASI and BSA, suggesting its retention in psoriatic lesions. A negative correlation was also observed between MG and CRP, AISI, and SII. These two inflammatory indices (AISI and SII), calculated based on blood morphology, are novel markers of systemic inflammation and immune status, and they are not directly related to CRP. This provides us with two independent ways to confirm that MG concentration in the serum of psoriasis patients correlates negatively with the intensity of systemic inflammation.

In the following section of the discussion, we will attempt to describe known alterations in processes related to MG that accompany psoriasis. Serum lactic acid concentration is increased in psoriatic patients. It is hypothesized that it might arise from accelerated glycolysis, which is required for rapid keratinocyte proliferation [27]. This observation is supported by increased expression of hexokinase [26]. Lactic acid is also a metabolite of methylglyoxal detoxification, as mentioned earlier. Glyoxalase 1 was found to be expressed in the red blood cells of psoriasis patients at the same level as in healthy individuals [28]. However, the concentration of glutathione (the cofactor essential for the proper activity of Glo1) is reduced in such patients due to increased oxidative stress [29]. Other enzymes that are able to detoxify MG are aldehyde dehydrogenase and aldose reductase [12]. The expression of aldehyde dehydrogenase is decreased in psoriatic lesions [26]. Therefore, seemingly, psoriasis, which is characterized by high oxidative stress, chronic inflammation, and impaired enzymatic systems, creates favorable conditions for MG accumulation.

However, these metabolic alterations are not the only ones observed during the course of the disease. Psoriasis patients also exhibit elevated levels of blood amino acids, possibly due to a rapid turnover of proteins in highly active keratinocytes. Among these are ornithine and citrulline, which are urea cycle intermediates. The amount of urea increases, too [27,30]. The activity of arginase I, which hydrolyses arginine to urea and ornithine, is higher, supporting cell proliferation [31]. Importantly, ornithine is a substrate for polyamine synthesis. Psoriatic lesions contain increased amounts of polyamines (putrescine, spermidine, and spermine) [32]. Spermidine and spermine are also elevated in blood [33], while putrescine and spermine are found in higher concentrations in urine [34]. Increased activity of ornithine decarboxylase, the enzyme responsible for polyamine synthesis, is also observed in psoriasis [35,36]. In summary, polyamines are synthesized at a high rate due to an abundance of substrates from accelerated amino acid turnover and the urea cycle.

It was observed that polyamines may bind to MG and inhibit the MG modification of proteins [37]. Therefore, polyamines may represent an additional mechanism, independent of previously described enzymatic systems, that regulates MG levels [38]. The decreased MG concentration in the serum of psoriasis patients observed in our study seems to be a consequence of the scavenging ability of polyamines. Additionally, polyamines, being polycations, can bind to nucleic acids. In animal models of psoriasis, it was observed that they assist RNA internalization by dendritic cells and self-RNA sensitization [39].

Although the patients in our study were not taking medications for psoriasis, it is worth exploring if it may affect MG levels. Methotrexate, a drug used in psoriasis therapy, inhibits Glo1 activity, promoting MG accumulation [40]. It is hypothesized that the increase in MG levels could be the mechanism of retinoid action in psoriasis [41]. Retinoid and glucocorticoid therapies reduce ornithine decarboxylase activity, leading to a decrease in polyamine levels, which aligns with our findings [35,42]. Furthermore, the amino acid levels in psoriasis patients respond to Etanercept (a biologic anti-TNFα drug), with their concentrations decreasing during therapy to levels similar to those seen in healthy individuals [30]. As mentioned previously, amino acids are substrates for polyamine synthesis. Based on these data, it looks like the aforementioned drugs act as factors that increase MG concentration. Their antiproliferative action may be mediated by MG. In fact, MG has been shown to have an antiproliferative effect on human cancer cells [43]. Our results emphasize the importance of further studies investigating MG as a potential marker of treatment response in psoriasis. However, due to contradictory data on MG concentration in the serum of psoriasis patients, its predictive value remains unclear. It was suggested as a prognostic factor in chronic kidney disease [44] and type 2 diabetes [45].

Given the limited data regarding MG in psoriasis, we could compare the results to other chronic diseases. MG (and other α-dicarbonyls) is not a reliable marker of protein glycation in systemic lupus erythematosus, possibly due to its high reactivity and fast turnover. Despite increased total concentrations of advanced glycation end-products (AGEs), MG is decreased in systemic lupus patients [2]. The same may be true for psoriasis, where a low MG concentration in the blood coincides with elevated AGEs in both the blood and skin [46,47]. In multiple sclerosis lesions, no alterations in MG and GO concentrations are detected, but MG-H1 (a major MG derivative) is present at an increased concentration [48]. In rheumatoid arthritis, highly immunogenic MG–protein adducts are detected [6].

AGEs may be formed and may accumulate in long-lived proteins, while substrates for their synthesis are rapidly depleted in inflammatory conditions [46]. Regardless of their source, AGEs accelerate inflammation and activate proinflammatory receptors. Maurelli et al. have discussed broader implications of AGEs in inflammatory diseases, providing additional support for our study’s significance [49]. Sultana et al. have highlighted the role of glyoxal-derived AGEs in skin inflammation [50]. Moreover, a decrease in AGE-related skin fluorescence is observed during the biological treatment of psoriasis [51].

In conclusion, it seems that MG is not a reliable marker of glycoxidation or inflammation intensity due to its high reactivity, possible retention in proteins as AGEs, and depletion through scavenging by polyamines. However, its negative correlation with disease severity and inflammation indices is worth further investigation in the context of disease monitoring. Additionally, the question arises if diets rich in polyamines, generally considered beneficial for health, would be beneficial in psoriasis.

## 5. Conclusions

Psoriasis is characterized by a reduced concentration of MG in blood serum, which additionally correlates negatively with lesion surface area, disease activity, and inflammatory indices and markers. The decreased concentration of MG appears to reflect its conversion to MG derivatives and the accumulation of these derivatives as AGEs in inflamed psoriatic skin and/or scavenging by polyamines. Further assessments of correlations between polyamines and MG concentration would enhance our understanding of these mechanisms. In future studies, it would be worth analyzing the nature of MG and polyamine reaction products and the possibility of the accumulation of such compounds in the tissues.

## Figures and Tables

**Table 1:** Characteristics of clinical and laboratory parameters of patients with psoriasis.

Examined Parameter	Mean ± SD	Median (Min–Max Range)
Body Surface Area (BSA) [%]	24.3 ± 17.5	19.0 (2.0–71.0)
Psoriasis Area and Severity Index (PASI)	13.8 ± 9.2	11.4 (3.2–43.8)
Dermatology Life Quality Index (DLQI)	14.2 ± 6.8	13.5 (2.0–26.0)
C-reactive protein (CRP) [mg/L]	5.8 ± 7.6	3.5 (0.5–35.2)
Leukocyte count [×10^9^/L]	7.9 ± 1.9	7.8 (4.6–11.6)
The Systemic Inflammation Response Index (SIRI)	2.1 ± 1.3	1.5 (0.3–5.9)
The Systemic Immuno-inflammation Index (SII)	843.5 ± 637.6	660.1 (187.9–3988.4)
The Aggregate Index of Systemic Inflammation (AISI)	556.9 ± 377.7	406.7 (73.3–1920.1)

SD—standard deviation.

**Table 2:** Correlations of MG concentration with analyzed clinical and biochemical parameters in patients with psoriasis.

Pair of Variables	*R*	*p*
MG [μg/mL] and BSA [%] ^2^	−0.32	**0.02**
MG [μg/mL] and PASI ^2^	−0.32	**0.02**
MG [μg/mL] and DLQI ^1^	−0.15	0.30
MG [μg/mL) and CRP [mg/L] ^2^	−0.45	**0.002**
MG [μg/mL) and Leukocyte Count [×10^9^/L] ^1^	−0.16	0.28
MG [μg/mL] and SIRI ^2^	−0.26	0.08
MG [μg/mL] and SII ^2^	−0.43	**0.003**
MG [μg/mL] and AISI ^2^	−0.37	**0.01**

^1^ Pearson’s test; ^2^ Spearman’s rank correlation test. Statistically significant results are in bold.

## Data Availability

The original contributions presented in this study are included in the article. Further inquiries can be directed to the corresponding author.
